# High-Quality Draft Genome Sequence of *Bacillus subtilis* Strain WAUSV36

**DOI:** 10.1128/genomeA.00586-16

**Published:** 2016-06-23

**Authors:** Jennifer Town, Patrice Audy, Susan M. Boyetchko, Tim J. Dumonceaux

**Affiliations:** a Saskatoon Research and Development Centre, Agriculture and Agri-Food Canada, Saskatoon, Saskatchewan, Canada; b Department of Veterinary Microbiology, University of Saskatchewan, Saskatoon, Saskatchewan, Canada; c Québec Research and Development Centre, Agriculture and Agri-Food Canada, Québec, Québec, Canada

## Abstract

*Bacillus subtilis* strain WAUSV36 inhibits the growth of and decreases disease symptoms caused by the potato pathogen *Phytophthora infestans*. We determined the sequence of the 4.7-Mbp genome of this strain. WAUSV36 shared very high nucleotide sequence identity with previously sequenced strains of *B. subtilis*.

## GENOME ANNOUNCEMENT

*Bacillus subtilis* WAUSV36 is a Gram-positive bacterium isolated from seeds of green foxtail harvested near Wauchope, Saskatchewan, Canada. This organism inhibits disease symptoms caused by the potato pathogen *Phytophthora infestans* in *in vivo* challenge assays and has been identified as a potential biocontrol agent for potato late blight.

*B. subtilis* WAUSV36 was grown at 22°C in a rotary shaker for 24 to 48 h in yeast extract glucose medium (2.0 g/liter yeast extract, 2.5 g/liter glucose, 0.4 mM MgSO_4_·7H_2_O, 0.09 mM MnSO_4_·H_2_O, 0.85 mM NaCl, 0.017 mM FeSO_4_·7H_2_O, 1.84 mM KH_2_PO_4_, and 1.43 mM K_2_HPO_4_). Genomic DNA was purified from 1 ml of overnight culture using the Wizard genomic DNA (gDNA) extraction kit (Promega, Madison, WI, USA) and sequenced on the GS Junior using the paired-end rapid library preparation protocol for Titanium chemistry (Roche, March 2012), with modifications as described previously (1). Reads from two paired-end sequencing runs (average read lengths of 418 and 419 bp) were assembled using Newbler version 3.0 (454 Life Sciences). The total number of filter-passed reads was 309,047. These reads were assembled into 2 scaffolds of 4,179,299 bp (19 contigs) and 59,592 bp (1 contig). The *N*_50_ contig size was 1,049,070 bp. Assembly of all sequencing data produced an improved high-quality draft (2) sequence featuring 25× genome coverage. Sequence data were annotated using the Prokaryotic Genome Annotation Pipeline version 3.1 (NCBI).

The genome size of *B. subtilis* WAUSV36 was 4,238,891 bp and was composed of 43.32% G+C content. A total of 4,510 genes and 4,404 protein-coding genes were observed, along with 2 genes encoding 5S rRNA, 3 genes encoding 16S rRNA, 5 genes encoding 23S rRNA, and 60 tRNA-encoding genes. A total of 1,688 Clusters of Orthologous Group (COG) clusters were identified by annotation using the Integrated Microbial Genomes (IMG) portal (https://img.jgi.doe.gov/cgi-bin/mer/main.cgi).

The sequences of taxonomic markers, such as the 16S rRNA-encoding gene and *rpoB* (3), were >99% identical to the corresponding sequences of many strains of *B. subtilis*. Similarly, the single-copy bacterial barcode marker *cpn60* (4) was identical in sequence to several strains of *B. subtilis*. At the whole-genome level, WAUSV36 had pairwise average nucleotide identities of 99.96% with 25 strains of *B. subtilis* available at the IMG portal and was below the specified nucleotide identity cutoff (5) for other species of *Bacillus*. Finally, SpecI (6) assigned WAUSV36 to the species cluster *B. subtilis*, with an average of 98.93% identity over 40 COGs. These observations suggest that WAUSV36 is a strain of *B. subtilis*.

Similar to other strains of *B. subtilis* associated with biocontrol phenotypes, the genome of WAUSV36 featured genes involved in biofilm formation (7), but no genes associated with surfactin production were observed. Five genes encoding putative beta-lactamases and three cellulase genes were also found.

### Nucleotide sequence accession numbers.

This whole-genome shotgun project has been deposited at DDBJ/ENA/GenBank under the accession no. LWLQ00000000. The version described in this paper is version LWLQ01000000.

## References

[1] Hill J, Chaban B, Town J, Links M, Dumonceaux T 2014 Modified paired end rapid library preparation protocol for 454 GS Junior 8-kb library preparation using Covaris g-tubes and BluePippin electrophoresis. Protoc Exch. doi:10.1038/protex.2014.028.

[2] Chain PSG, Grafham DV, Fulton RS, Fitzgerald MG, Hostetler J, Muzny D, Ali J, Birren B, Bruce DC, Buhay C, Cole JR, Ding Y, Dugan S, Field D, Garrity GM, Gibbs R, Graves T, Han CS, Harrison SH, Highlander S, Hugenholtz P, Khouri HM, Kodira CD, Kolker E, Kyrpides NC, Lang D, Lapidus A, Malfatti SA, Markowitz V, Metha T, Nelson KE, Parkhill J, Pitluck S, Qin X, Read TD, Schmutz J, Sozhamannan S, Sterk P, Strausberg RL, Sutton G, Thomson NR, Tiedje JM, Weinstock G, Wollam A, Genomic Standards Consortium Human Microbiome Project Jumpstart Consortium, Detter JC 2009 Genome project standards in a new era of sequencing. Science 326:236–237. doi:10.1126/science.1180614. 19815760 PMC3854948

[3] Adékambi T, Drancourt M, Raoult D 2009 The *rpoB* gene as a tool for clinical microbiologists. Trends Microbiol 17:37–45. doi:10.1016/j.tim.2008.09.008. 19081723

[4] Links MG, Dumonceaux TJ, Hemmingsen SM, Hill JE 2012 The chaperonin-60 universal target is a bar code for bacteria that enables *de novo* assembly of metagenomic sequence data. PLoS One 7:e49755. doi:10.1371/journal.pone.0049755. 23189159 PMC3506640

[5] Richter M, Rosselló-Móra R 2009 Shifting the genomic gold standard for the prokaryotic species definition. Proc Natl Acad Sci USA 106:19126–19131. doi:10.1073/pnas.0906412106. 19855009 PMC2776425

[6] Mende DR, Sunagawa S, Zeller G, Bork P 2013 Accurate and universal delineation of prokaryotic species. Nat Methods 10:881–884. doi:10.1038/nmeth.2575. 23892899

[7] Bais HP, Fall R, Vivanco JM 2004 Biocontrol of *Bacillus subtilis* against infection of *Arabidopsis* roots by *Pseudomonas syringae* is facilitated by biofilm formation and surfactin production. Plant Physiol 134:307–319. doi:10.1104/pp.103.028712. 14684838 PMC316310

